# The Out-Patient System and Its Abuse

**Published:** 1896-02-29

**Authors:** 


### The Institutional Workshop.

### THE OUT-PATIENT SYSTEM AND ITS ABUSE.

Those who read the report which appeared in The Hospital of Feb. 8th of the meeting of the Council of the Hospital Sunday Fund will not have failed to notice the discussion which was raised on the out-patient question by the motion of Dr. Glover to refer the matter to a committee for investigation. In fact, we cannot be too thankful to those who take the trouble again and again to draw attention to the ever-growing incubus of out-patient and casualty cases, by which the work of our hospitals is being degraded, and their reputation, as doers of good, is gradually being undermined in consequence of the conviction growing in the minds of many that, by the pauperising influence of their vast out-patient system, they are doers of evil also. We are glad that the matter has been put in the hands of the Distribution Committee, for, while we do not think that the Fund can usefully, or even safely, undertake the general duty of supervising the whole internal economy of the London hospitals, we cannot shut our eyes to the fact that a committee with money to distribute comes to the rectification of a great abuse with much more influence behind it than do those who merely feel constrained to give vent to pious opinions on the subject.

Our inquiries into this matter have shown that the out-patient system, as at present worked in connection with most of our great hospitals, is not the real benefit to the deserving poor which it ought to be, that it is a distinct and grievous injury to general practitioners, and that it is a great pauperising influence, gradually drawing into its net people who otherwise would never dream of accepting eleemosynary aid. But they have also enabled us to see that there are two main influences which tend to keep this system on its feet, each of which will require very different treatment. On the one hand it is fairly plain that one great reason, from the financial and secretarial point of view, for encouraging a large out-patient department is that it is a huge advertisement. In the present temper of the public in regard to payment by results the most effectual appeal to the man in the street is thought to be by a startling statement of results and by a great parade of figures. This is a view with which we have but small sympathy, but of its potency in keeping up the out-patient system we are sure. From the chairman and the secretary down to the porter who enters the cases at the gate all strive to run up the figures, so as to stand well with the subscribing public by showing the vast good the hospital is doing.

The other influence to which we allude is that of the medical staff. Notwithstanding the wailing and lamentation which arises from the rank and file of the medical profession in regard to out-patient abuse, we believe that many of the real difficulties which surround and embarrass every committee which endeavours to solve this problem arise from the attitude of the medical men connected with the hospitals. We do not blame them. We merely state a fact which is well worthy of the attention of those other medical men who are outside these institutions.

Hospital physicians and surgeons are but men. They are brimful of human nature; like others of their race are actuated by ordinary human motives; and seeing that they are not paid a penny for their services they struggle for their reward in other forms. The reward they seek is an honourable one, and takes the form of experience and fame. But to gain these ends they must see many cases. It is by the careful treatment and laborious observation of certain types of disease that each man seeks for fame and fortune. To find such cases he willingly undergoes the drudgery involved in out-patient work, and searches through enormous shoals of patients for the sake of a small residuum of "cases." The social position of the patients is nothing to him, except that better-class patients being cleaner are more pleasant to deal with.

The out-patient physician or surgeon has no say in the management or finances of the hospital, and very often he has but little touch with the general practitioner, for the days of consultation have not yet come, so he works on ploddingly, piling up a great experience and giving out his best powers in teaching the students of to-day, the practitioner of the future; and in thus acting casts his bread upon the waters. And who shall blame him for so doing? He hopes that he is founding a career, but at the same time it is by the laborious care which he bestows upon his patients that the fame also of the hospital is extended. All this, however, tends to increase the out-patient difficulty. The very basis of fame, both for self and hospital, is a vast crowd of out-patients, who may be daily passed through the sieve; and hence, we think, arises much of the passive resistance on the medical side which blocks the way of such reformers as are willing to intrude on this most difficult question. We would ask then in all seriousness whether medical men, harassed as they are by the ceaseless growth of the out-patient system, will not make some organised endeavour to diminish the nuisance of which they complain? The medical staff of every large hospital is a little world in itself, and every member is largely influenced by the opinion of his fellows. What is wanted is to encourage, in each individual of this little world, a strong feeling in favour of restricting the out-patient department at his own particular hospital, and this we fancy can only be done by a definitely expressed and organised movement in favour of consulting with those men who are connected with hospitals which deal properly with this out-patient question.

The centre of the whole question is that it should be strongly and practically borne in upon the most influential members of the medical staffs of the various hospitals that the medical profession is in favour of restrictions being placed upon the extension of their out-patient departments, and that in proportion as these are extended, so consultations will flow in other directions.

It is in all friendliness, and with every respect for the motives which underlie the support given by hospital doctors to the present system, that we give the hint and make the suggestion that some change in their attitude is desirable. So far as the financial and secretarial side of the question is concerned, we think the Distribution Committee of the Hospital Sunday Fund, which occupies the position of an almoner, will be able to bring much influence to bear in the direction of reform. It lies with the medical profession, in its turn, to bring its influence to bear in the same direction, and for every man to force on the attention of the members of the staff of the hospital where he was educated, with whom he naturally consults the most, that in future consultations will go where out-patients are properly discriminated. If they cannot do this we shall have but slight faith in the *bonâ fides* of their complaints.

